# Editorial

**Published:** 1849-01

**Authors:** 


### THE MEDICAL EXAMINER.

### PHILADELPHIA, JANUARY, 1849.

A CARD.

The late Editor of the Medical Examiner, in so suddenly retiring from its management, deems it respectful to its patrons to offer a few words of explanation.

To many it is known that his editorial duties have been performed under great disadvantages. More than thirty years of uninterrupted devotion to the practice of his profession, has naturally brought upon him the labour and distractions incident to an extensive business; these, with his public duties and his domestic affairs, have rendered it impossible for him to give to the journal the attention demanded by its importance and his own reputation. In the last number, he adverted briefly to this subject, and expressed a hope that, in future, he should be able to make arrangements more in accordance with his wishes; further reflexion, however, has satisfied him that it is better for him to relinquish the task, and devote himself more exclusively to his other imperative duties. In taking this step, he is happy in the opportunity of giving place to gentlemen every way capable of maintaining the character and usefulness of the journal. The present editors, are extensively and favourably known as teachers and authors, and as the editorial labours will be divided, the task to them individually will be less onerous, whilst the interest of the journal will be promoted by the increased attention they will be able to bestow upon it. Their characters are a sufficient guarantee that it will be conducted with ability and independence, whilst their disconnection with any of the Colleges will protect them from the ascription of wrong motives in their impartial criticisms. Hoping that the liberal support accorded to the Medical Examiner in past years will be continued under its new conductors, and that all parties will be benefitted by the change, with many thanks to its readers for their generous indulgence, he now bids them *adieu*.

---

It will be seen by the above card, that the Medical Examiner has passed from the management of the former able editor into the hands of those whose names appear upon the cover. In assuming the responsible position of editors of a Medical Journal, so widely circulated, and lately so successfully conducted, the present incumbents feel that they will have to draw largely upon the kindness and indulgence of its patrons. The duties have devolved upon them so suddenly and unexpectedly, that they have scarcely had time to make the necessary arrangements incident to a change, before the day of appearance of the Journal had arrived. This, it is trusted, will prove a sufficient apology for the scarcity of original articles and notices in this number.

In conducting the Journal, the present editors desire and intend, as far as lieth in them, to be strictly independent and impartial, with the earnest hope that no wrong motive or sectional prejudice may be attributed to whatever may appear from them in their official capacity. It will be observed, that in the present number the arrangement of the Record is somewhat altered, each department being classified under its appropriate head; this, it is hoped, will add to its interest, and enable the reader of the Examiner readily to find that which is most interesting and useful to himself. The large supply of foreign and domestic periodicals received by this Journal, will enable the editors to present the most recent information in every department of Medical Science. They also propose to make arrangements for the reception of reports from Practitioners, Hospitals, and Cliniques, of interesting cases; by these means, trusting to enhance the value of their Journal.

The readers of the Examiner, and the profession generally, will confer a favour upon the Editors by contributing to the pages of their Journal whatever may be of interest in any department of Medical Science.

---

PROGRESS OF THE CHOLERA IN THE UNITED STATES.

For the purpose of presenting our readers with the latest account of the progress of the cholera in New York, we extract from the columns of the "Courrier des Etats Unis," the following communication from M. le docteur Bodinier, ex-chirurgien interne des hôpitaux de Paris—docteur en mèdecine et docteur en chirurgie de la Faculté de Paris:

"The ship 'New York' left Havre, in which port no cholera exists, on the 9th November, 1848. There were on board, including passengers and crew, 385 persons, in the enjoyment of good health. The voyage was favourable, in every respect, during the first sixteen days. At this time the vessel had arrived near the shores of Nova Scotia, (lat. 49°, long. 61°,) when a passenger in the steerage, aged 29 years, was taken sick. This was a case of cholera. The second case occurred two days after, in a man 62 years of age; the third and fourth in two children 3 years old; the fifth in a man 40 years of age, and the sixth and seventh, in two children five years old. All of these died between Nov. 29, and Dec. 3d, the day on which the ship entered the port. Twelve other sick persons were on board, and were landed at the Quarantine with the rest of the passengers.

Three days after the arrival of the ship, six new cases of cholera made their appearance, four of these occurred in persons at the hospital before the arrival of the 'New York.'

At the same time, Dec. 5th, a convalescent person, not one of the passengers of the New York, came to the city. He was attacked with the cholera in the night, and carried back to the Quarantine. Some new cases showed themselves at the hospital on the 7th, 8th and 9th of December, among the passengers of the New York, and two others among those in the hospital previous to the arrival of the vessel. The weather on the 10th, 11th and 12th of December, being mild and dry, the epidemic appeared to cease. During two days there were no new cases. On the 13th, 14th and 15th of December, the weather became damp and cloudy, and thirteen new cases appeared among the passengers in the steerage of the New York, who had been retained at the Quarantine. The ship, after twelve days' quarantine, came to the wharf with her crew and cabin passengers, none of whom had as yet been sick. Thus, up to this time, the epidemic had attacked, whether on board or at the Quarantine, *only* the steerage passengers, both French and Germans, coming from regions of country where there had been no cholera previous to their departure, and who could not have been infected by the contagion communicated from one person to another.

On the 10th, at the moment when the epidemic appeared to have ceased at the hospital, a German, living in the lower part of the city, in the house where the cholera patient had passed the night, and who had not been at the Quarantine, was attacked with cholera. It was in a large room with six or seven beds, and only one of those, who slept there, was attacked. The patient died, and the physicians who saw him, assert that it was a case of Asiatic Cholera. There has not, however, been any other case, either in the house alluded to, or in the city. So that this fact, which at first sight would seem to give great weight to the belief 'that the disease is contagious,' consequently exciting unnecessary alarm, has, on the contrary, only the value of a simple coincidence; if attention is paid to the fact, that there were a great number (perhaps more than fifteen persons,) who slept at the same time in the same room, without being sick, more than other persons of the house, all of whom, on account of the crowded apartment, of the regimen, and of the want of cleanliness, are as much predisposed as possible to the developement of the scourge which is threatening us.

In a word, there has been, up to the present time, about fifty cases of cholera at the Quarantine. It is the true Asiatic disease which exists there. I have seen various types of the disease among those who are still sick. But that which should restore the public confidence, is that the treatment employed, when applied in time, has succeeded in saving nearly all the cases, in spite of the enfeebled condition of the poor emigrants, who, during a long voyage, are crowded in the steerage and subjected to all the influences which are so favourable to the developement and malignity of the cholera."

In addition to the above interesting account, we learn from our daily papers' reports, that on Monday, the 18th ult., five new cases of cholera occurred at the Marine Hospital. Since Sunday at noon, and within the same period, there have been three deaths. On Tuesday afternoon, the Health Officer at the Quarantine reports two new cases and two deaths, since the report of Monday. On Thursday, several are reported to have died, who were inmates of the Hospital previous to the arrival of the ship New York.

The only account we have of its further progress in the city, is the following:

"CHOLERA IN NEW YORK CITY.—The resident physician reports a case of cholera as having occurred on Wednesday morning, at 161 Washington street, N. Y., at a German boarding house. The case is that of a German who has been twelve months in the United States—arrived from Pittsburg here eight days since—has had no intercourse with the passengers of the ship New York, and had taken passage in one of Fox & Livingston's vessels, and would have sailed that day for Europe if this attack had not prevented him. He died during the day."

The cholera has not made its appearance either in this city or at the Quarantine. In Baltimore the following report has reached us:—

"Considerable excitement has prevailed in our community during the afternoon, growing out of a report that the cholera had actually made its appearance in our city, that several deaths have taken place, &c. After careful examination I find that the report had its origin in the following circumstances:

The ship Cyrus Richards, Capt. Welsh, arrived here to-day from Rotterdam. It appears that during the passage several cases of cholera occurred. The captain was among the first attacked, but by timely assistance and the proper remedies, he recovered. Mr. Baines, the first mate, one of the seamen, and one of the passengers, were next attacked, and notwithstanding every attention was paid to them, the three died. Several others were attacked, but they fortunately escaped.

The last case occurred on the 19th of November, and on the 25th the officers, crew, and passengers were in the enjoyment of perfect health. The ship is now at quarantine strictly guarded. She has been visited by the Health Officer, who reports that there is not a case of cholera on board."

Through the telegraphic reports, we learn that this disease has made its appearance in New Orleans; five deaths are reported as having occurred on the 16th inst.

WESTERN JOURNAL OF MEDICINE AMD SURGERY.

It is gratifying to us, at the very commencement of our career, to be spared the discouraging prospect of want of success in an ably conducted Journal. By the last number (December) of the Western Journal of Medicine and Surgery, we learn that the prompt response given to the appeal of the editors in the November number, has decided them to continue the work. May success attend their renewed exertions.

NAVAL APPOINTMENTS.

The following Assistant Surgeons in the Navy, examined by the Medical Board recently convened at the Naval Asylum, Philadelphia, have been found qualified for promotion, and passed, viz:

Andrew A. Henderson, Passed Assistant Surgeon, to rank next after Passed Assistant Surgeon J. Hopkinson.

Elisha K. Kane, Passed Assistant Surgeon, to rank next after Passed Assistant Surgeon J. Wilson, Jr.

Edw. Hudson, Passed Assistant Surgeon, to rank next after Passed Assistant Surgeon E. K. Kane.

Of the candidates examined for admission into the service as Assistant Surgeons, the following have been found qualified:

Francis M. Gunnell, of the District of Columbia; Jas. Suddards, of Pa.; Robert Carter, of Va.; S. Allen Engles and Edward Shippen, of Pa.; Gerard Alexander, of Ky.; Benjamin Vreeland, of N. Y.; Walter Hore, Carthon Archer, Richard B. Tunstall and Charles H. Williamson, of Va.; James F. Heustis, of La.; Arthur M. Lynah, of S. C.

The candidates will inform the Department of their respective places of residence.

*Navy Department*, Dec. 7, 1848.

MANSLAUGHTER BY VACCINATION.

A physician of Pulaski county, Illinois, has been sentenced to the penitentiary for the term of four years and a half, upon a charge of manslaughter, growing out of his vaccinating a man with small-pox matter from the effects of which he died. Particulars not given.

